# Resolution of Inflammation in Retinal Disorders: Briefly the State

**DOI:** 10.3390/ijms23094501

**Published:** 2022-04-19

**Authors:** Maria Consiglia Trotta, Carlo Gesualdo, Francesco Petrillo, Caterina Claudia Lepre, Alberto Della Corte, Giancuomo Cavasso, Giulia Maggiore, Anca Hermenean, Francesca Simonelli, Michele D’Amico, Settimio Rossi

**Affiliations:** 1 Department of Experimental Medicine, University of Campania “Luigi Vanvitelli”, Via Santa Maria di Costantinopoli 16, 80138 Naples, Italy; mariaconsiglia.trotta2@unicampania.it (M.C.T.); francesco.petrillo@unicampania.it (F.P.); caterinac.lepre@gmail.com (C.C.L.); michele.damico@unicampania.it (M.D.); 2 Eye Clinic, Multidisciplinary Department of Medical, Surgical and Dental Sciences, University of Campania “Luigi Vanvitelli”, Via Luigi De Crecchio 6, 80131 Naples, Italy; carlo.gesualdo@unicampania.it (C.G.); albertodellacorte@live.it (A.D.C.); giancuomo.cavasso@studenti.unicampania.it (G.C.); francesca.simonelli@unicampania.it (F.S.); 3 Department of Ophthalmology, University of Foggia, Viale Luigi Pinto 1, 71122 Foggia, Italy; giuliamaggiore@icloud.com; 4 “Aurel Ardelean” Institute of Life Sciences, Vasile Goldis Western University of Arad, 86 Revolutiei Av., 310414 Arad, Romania; anca.hermenean@gmail.com

**Keywords:** retinopathy, inflammation, pro-resolving mediators, lipoxins, resolvins, protectins, maresins, annexins, galectins

## Abstract

The most frequent retinal diseases, such as diabetic retinopathy, age-related macular degeneration and posterior uveitis, are underlined by oxidative stress or aging-induced retinal inflammation, which contributes to vision impairing or loss. Resolution of inflammation is emerging as a critical phase able to counteract the inflammatory process leading to the progression of retinal damage. Particularly, pro-resolving mediators (PMs) play a key role in the modulation of inflammatory exudates and could be considered a new target to be investigated in different inflammatory-autoimmune pathologies. Here, we highlight the most recent studies concerning the role of the main PMs (lipoxins, resolvins, prtectins, maresins and annexins) in retinal inflammation, in order to collect the best evidence in the field of inflammatory retinal damage resolution and to propose novel pharmacological approaches in the management of the most common retinal diseases.

## 1. Inflammation in Retinopathies

Retinal inflammation is a predominant factor contributing to the pathogenesis of the most common retinal diseases, such as age-related macular degeneration (AMD), posterior uveitis and diabetic retinopathy (DR) [1].

In this latter, the increase of pro-inflammatory mediators is a direct consequence of the metabolic changes due to chronic hyperglycemia. DR can be divided into two stages: non-proliferative (NPDR) and proliferative DR (PDR). In NPDR, chronic hyperglycemia and the underlying chronic inflammation cause progressive changes in the retinal capillary microcirculation with the consequent appearance of hemorrhages, microaneurysms and intraretinal exudates. In contrast, the PDR represents the late stage of the retinopathy, characterized by the presence of numerous ischemic retinal areas that determine an increase in the production of vascular endothelial growth factor (VEGF), with consequent growth of retinal new vessels [2]. This stage is underlined by inflammatory processes too, which lead to occluded and degenerated capillaries [3]. These latter, under the stimulus of the pro-inflammatory angiogenic cytokines, such as interleukin 8 (IL-8) and tumor necrosis factor alpha (TNF-α), promote proliferation of the endothelial cells and progression of a fibrovascular-related retinal fibrosis typical of DR [4].

Chronic inflammation is crucial for AMD and is induced by age-related oxidative stress that leads to loss of retinal pigment epithelium (RPE) and photoreceptors function [3]. The dry form of AMD is characterized by the accumulation of drusen in the subretinal space, which over time can lead to progressive photoreceptor degeneration. They contain numerous potentially harmful constituents including lipids, lipoproteins and cell debris derived from RPE, along with numerous factors linked to inflammation, such as complement components, immunoglobulins and acute phase proteins such as fibrinogen (Fg), α1-antichimotrypsin (AAT), vitronectin (VTN) and pentraxin (PTX). Furthermore, it has been shown that the aforementioned material contained in the drusen is pro-inflammatory through the activation of some pathways such as nuclear factor-kappa-light-chain-enhancer of activated B cells (NF-κB) and the inflammasome [5,6,7].

In contrast, wet AMD is characterized by excessive amounts of VEGF produced from RPE that contributes to the breakdown of the external blood-retinal barrier and the germination from choroid of fragile newly formed blood vessels into the retinal tissue [8,9].

Concerning uveitis, lymphocytes resident in ocular tissues and the related cytokine pattern play an important role in the initiation and progression of ocular inflammation [10]. In fact, there are many studies on autoimmune and endotoxin-induced uveitis (EIU) animal models, as well as studies on aqueous, vitreous and serum of patients with uveitis, which show a central role of Th17 cells and related pro-inflammatory cytokines such as interleukin 6 (IL-6), interleukin 10 (IL-10), interleukin 17 (IL-17), interleukin 22 (IL-22), interleukin 23 (IL-23) and TNF-α in the pathogenesis of uveitis itself [11].

Overall, anti-inflammatory therapies could potentially prevent or modify the course and the severity of retinal disorders [12] Similarly, therapies based on the use of endogenous PMs appropriately formulated for exogenous use or the comprehension of the whole damaging mediators and mechanisms to target in retinopathies could help future medicine for retinal diseases. Therefore, the goal of this review is to highlight the latest evidence in DR, AMD and posterior uveitis.

## 2. Pro-Resolving Mediators in Retinopathies

**Figure 1:**
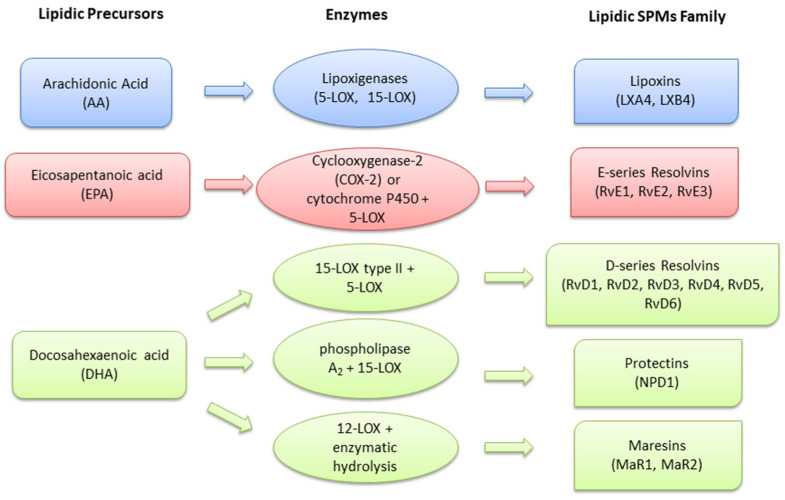
Main lipidic PMs: precursors and biosynthesis enzymes. LOX: lipoxygenase; COX: cyclooxygenase.

PMs are local mediators of a lipidic nature, derived from omega-6 and omega-3 polyunsaturated fatty acids (PUFAs). They are converted into potent PMs through the action of specific enzymes, such as cyclooxygenase (COX) and lipoxygenase (LOX), and are aimed at resolution of the inflammation in a tissue [12,13] (Figure 1).

The first class of PMs described were lipoxins (LXs). They are biosynthesized from the omega-6 PUFA arachidonic acid (AA) through 5-LOX and 15-LOX (LXA4, LXB4, Epi-Lipoxin A4, Epi-Lipoxin B4) and exert their effects by binding the G protein-coupled lipoxin A4 receptor (ALX)/formyl peptide receptor (FPR2) with high affinity [14,15], and other receptors, such as, for example, G protein-coupled receptor 32 (GPR 32), Aryl hydrocarbon receptor, high affinity cysteinyl leukotriene receptor and estrogen receptor [15]. Resolvins (Rvs), protectins (PDs) and maresins (MaRs) are all PMs converted from the omega-3 PUFAs eicosapentaenoic acid (EPA) and docosahexaenoic acid (DHA) [13]. Specifically, Rvs are classified as E-series (RvE1, RvE2 and RvE3) if they derive from EPA and as D-series (RvD1, RvD2, RvD3, RvD4, RvD5, RvD6, AT-RvD1, AT-RvD2, AT-RvD3 and AT-RvD4) if they are generated from DHA [13,16,17,18]. RvD1 and RvD3 bind preferentially to the ALX/FPR2 receptor, RvD2 acts on G-protein-coupled receptor 18 (GPR18) and RvE1 interacts with the G protein-coupled receptor BLT1 [19]. Lastly, protectin D1 (PD1), maresins 1 and 2 (MaR1, MaR2, MCTR1 and MCTR2) derive from DHA [13,20]. Less is known about their specific receptors: MaR1 has been recently reported to bind the G protein–coupled receptor 6 (LGR6) [21], while neuroprotectin D1 (NPD1) seems to selectively bind some specific receptors in RPE cells [22]. A specific glucocorticoid regulated protein, called Annexin A1 (AnxA1), is considered an important anti-inflammatory and PM [23], which shares the ALX/FPR2 receptor as its receptor, together with LXA4, RvD1 and RvD3 [24]. Through the bond with the ALX/FPR2 receptor, AnxA1 is able to reduce the infiltration of neutrophils in the inflammatory site and, consequently, to reduce the release of pro-inflammatory cytokines [25].

### 2.1. Lipoxins

Both LXA4 and LXB4 are reported to be synthesized in the inner retina, with their levels decreased following acute or chronic injury [26,27]. However, a higher number of data are reported on LXA4 compared to LXB4. LXA4 retinal properties have been extensively investigated: besides reducing IL-6 content in human Adult Retinal Pigment Epithelial cell line-19 (ARPE-19) exposed to lipopolysaccharide (LPS) [28], LXA4 was able to delay the progression of retinal degeneration. Indeed, in RD1 mice, a model of rapid retinal degeneration, LXA4 byosintesis (5-LOX and 15-LOX) and receptor (ALX/FPR2) resulted in markedly downregulated in end-stage RD1 retinas [29]. Interestingly, when intravitreally injected in RD1 mice, LXA4 restored visual function by reducing the apoptosis of Photoreceptor Outer Segment (POS) cells through the inhibition of pro-inflammatory gene expression and the modulation of microglial activities [29]. This evidence was recently confirmed in a mouse model of AMD: Balb-c mice, orally treated with LXA4 and then exposed to oxidative-stress injury induced by blue-light exposure, exhibited a reduced retinal damage in terms of Outer Nuclear Layer (ONL) thickness and tight junctions [30]. LXA4 also reduced reactive oxygen species (ROS) and consequent death in RPE cells exposed to blue-light [30].

LXA4 seems to also have an important role in delaying the progression of DR: both patients with NPDR or PDR showed significantly reduced LXA4 serum levels compared to diabetic subjects without retinopathy [28]. Moreover, LXA4 vitreous levels were also significantly decreased in PDR patients [28].

The pharmacological activation of the LXA4 signaling pathway has also been shown to potently reduce EIU in rats by decreasing the number of inflammatory cells and protein dispersion in the aqueous humor. This effect was associated with reduced production of interleukin 1 beta (IL-1β), TNF-α, prostaglandin-E2 (PGE2), and expression of COX-2, VEGF, NF-kB and c-Jun. It is very interesting that an anti-inflammatory effect was achieved even when LXA4 was applied topically after LPS administration, with an efficacy similar to topical treatment with prednisolone [31].

Therefore, by further investigating LXA4 and LXB4 pathways and eventual crosstalks (Table 1), these PMs could be considered for the prevention and treatment of various retinal disorders.

### 2.2. Resolvins

Rvs are a class of endogenous lipid-derived molecules, locally produced from EPA (through COX-2 and 5-LOX) or DHA (through 15-LOX and 5-LOX), with powerful immunomodulating properties. Indeed, Rvs are able to block hyperactivated immune responses, by acting as a “stop” signal in inflammatory processes, which otherwise would become pathological [16]. Since 2002, when Serhan et al. introduced the term “resolvins” for these mediators to indicate their ability to downregulate leukocyte exudation cells and to regulate the timing in the resolution of inflammation [16,32], several pre-clinical and clinical studies have been performed to investigate Rvs properties in disorders with immune-inflammatory pathogenesis, such as autoimmune diseases, rheumatoid arthritis, cardiovascular diseases and ocular diseases [33,34,35] such as dry-eye. Unfortunately, an important problem of Rvs is the systemic metabolic stability of the product which conditions their use in ophthalmology. Serhan and Rashid have widely demonstrated in the dry-eye that this is a problem that can be easily solved by the topical administration of ω-3 fatty acids [35,36]. Indeed, α-linolenic acid eye drops, available as a microemulsion of PUFAs and moisturizing polymers, could act as a reservoir for the localized Rvs biosynthesis [36,37].

There are few pre-clinical studies conducted so far on the possible role of Rvs in retinopathies (Table 2). A protective action exerted by RvD1 in retinal aging has been proposed by Trotta et al. [38], reporting a significant reduction in endogenous RvD1 retinal levels in aged Balb-c mice compared to younger mice. Particularly, these were less reduced in aged females compared to aged males. Moreover, endogenous RvD1retinal levels showed a significant inverse correlation with retinal microglia and astrocyte activation, neuroinflammation, apoptosis and nitrosative stress [38].

RvD1 has also been shown to activate the ALX/FPR2 receptor in primary retinal photoreceptor cells exposed to high glucose, as a model of DR in vitro [39]. Consequently, RvD1 was able to reduce the high-glucose induced mitochondrial damage through a restoration of mitochondrial morphology and function, which lead to an increased survival of primary retinal photoreceptor cells, promoted by RvD1 survival [40]. Moreover, RvD1 decreased the levels of VEGF and pro-angiogenetic microRNAs (miRNA), such as miR-20a-3p, miR-20a-5p, miR-106a-5p, and miR-20b. This occurred not only in primary retinal photoreceptors, but also in the exosomes released by these cells, suggesting a role of RvD1 in the modulation of neovascularization process [39].

The effects of RvD1 were also tested by Rossi et al. in an EIU mouse model with retinal involvement [41]. The authors observed that RvD1 intravitreal injections significantly and dose-dependently reduced the clinical score attributed to endotoxic uveitis, an effect mediated by a reduced presence of B and T lymphocytes in retinal tissue, along with a decreased presence of pro-inflammatory M1 macrophages and a concomitant greater presence of anti-inflammatory M2 macrophages in neuroretinal layers [41]. In the same animal model, Rossi et al. reported an interaction between intravitreal RvD1 and local endogenous sirtuin-1 (SIRT1) [42], a protein with nicotinamide adenine dinucleotide (NAD+)-dependent histone deacetylase activity, whose dysregulation has been linked to several retinal diseases [43].

In line with RvD1 are the data concerning RvE1. It seems to have a protective role in retinal neovascularization. Tian H et al. tested the effects of RvE1 and an analog RvE1 (RX-10008), intraperitoneally administered, in a mouse model of laser-induced choroidal neovascularization (CNV) provided by a diode laser. The authors observed a reduction in losses in the first week, suggesting that RvE1 protected by acting on early events, unlike the analogue RX-10008, whose effect approached that of RvE1 only around day 14. The amplitude of the CNV was reduced by 70% with RvE1, but remained unchanged with the analog, thus suggesting that RvE1 may be more efficient than the analog in reducing the size of the CNV [44].

Subsequently, Haibin et al. studied the biosynthesis of RvE1 and RvD1 in choroid-retinal endothelial cells (CRECs) and in leukocytes under inflammatory conditions. Inflammatory stimulation increased the biosynthesis of RvE1 and RvD1 from EPA and DHA, respectively, in CREC and leukocyte coculture. RvE1 and RvD1 reduced expression of vascular cell adhesion molecule-1 (VCAM-1), IL-8, inflammatory macrophage protein-1 beta (MIP-1β) and TNF-α from CRECs or coculture of CRECs and leukocytes. Finally, treatment of CRECs or polymorphonuclear leukocytes (PMN) with RvE1 or RvD1 inhibited PMN transmigration across the CREC barriers [45].

Both RvD1 and RvE1 exhibited a protective role in a mouse model of oxygen-induced retinopathy (OIR). This model is characterized by vascular loss and regrowth, with hypoxia-induced pathological neovascularization [45].

Overall, the above mentioned Rvs are potential targets and tools in the meantime for the novel treatment of retinal diseases, acting as an early counter-regulator of the pathways underlying the dysfunction of retinal cells.

**Table 2:** Resolvins and retinal damage.

Author, Year	Model	Treatment	Main Results
Connor et al., 2007 [46]	In vivo	C57BL/6J mice fed with diet containing 2% ω-6-PUFAs (AA) and no ω-3-PUFAs (DHA and EPA), or 2% ω-3-PUFAs and no ω-6-PUFAs in 10% (*w/w*) safflower oil	Among the bioactive ω-3-PUFA-derived mediators, RvD1 and RvE1 potently protected against neovascularization
	C57BL/6J mice exposed to 75% O_2_ from postnatal day 7 (P7) to postnatal day 12 (P12), then returned to room air for 5 days as a model of hypoxia-induced ischemic retinopathy		
Tian et al., 2008 [44]	In vivo	Intraperitoneal injections of RvE1 (18.7 µg/kg) and RX-10008 (RvE1 analog, 14.3 µg/kg) on days 1, 2, 4, 6, and 8 after CNV induction. Evaluation after 14 days	RvE1 reduced leakage and choroid lesion starting from day 7, while RvE1 analog was efficient at day 14
	Laser rupture of Bruch’s membrane, as a CNV model		
Tian et al., 2009 [45]	In vitro	Cells stimulated with 50 nM RvE1 or RvD1 for 8 h before IL-1β exposure	RvD1 and RvE1 biosynthesis was stimulated in CRECs co-cultured with lymphocytes under inflammatory stimulus. RvD1 and RvE1 reduced pro-inflammatory mediators such as VCAM, IL-8, MIP-1β and TNF-α. Moreover, RvD1 and RvE1 reduced PMN transmigration across the CREC barrier
	CRECs cultured alone, in co-culture with lymphocytes or PMN. Cells exposed to 2 ng/mL IL-1β for 4, 12 and 24 h		
Rossi et al., 2015 [41]	In vivo	Intravitreal injections (5 µL) of RvD1 (10–100–1000 ng/kg) into the right eye 1 h following LPS treatment. Evaluation after 24 h	The EIU score was significantly and dose-dependently decreased by all 3 doses of RvD1. Retinal layers showed a reduced presence of B and T lymphocytes. Neuroretinal layers exhibited a decreased staining of pro-inflammatory M1 macrophages and a concomitant increment of anti-inflammatory M2 macrophages
	Rat EIU with retinal involvement, induced with 200 μg of LPS into the footpad of Sprague-Dawley rats		
Rossi et al., 2015 [42]	In vivo	Intravitreal injections of RvD1 (10–100–1000 ng/kg) into the right eye 1 h following LPS treatment; intravitreal injections of the ALX/FPR2 inhibitor Boc-2 (0.4mg/kg/4 μL) 30 min before RvD1 1000 ng/kg. Evaluation after 24 h. Daily intraperitoneal injections of SIRT1 inhibitor EX527 (10mg/kg/day) for 7 days prior EIU, followed by RvD1 (1000 ng/kg), intravitreally injected 1 h following LPS. Evaluation after 24 h.	SIRT1 expression was dose-dependently increased by RvD1. The inhibition of ALX/FPR2 receptor decreased SIRT1 expression. The effects of RvD1 (1000 ng/kg) were partly abolished by the inhibition of SIRT-1 activity
	Rat EIU with retinal involvement, induced with 200 μg of LPS into the footpad of Sprague-Dawley rats		
Maisto et al., 2020 [39]	In vivo	RvD1 (50 nM) alone or combined with the ALX/FPR2 inhibitor Boc-2 (20 µM) added to primary retinal photoreceptor cells at the time of adding high glucose	High glucose increased VEGF levels in photoreceptors and their released exosomes, with a decrement of cellular and exosomal anti-angiogenic miR-20a-3p, miR-20a-5p, miR-106a-5p and miR-20b. By activating ALX/FPR2 receptor, RvD1 reverted the effects of high glucose on both primary retinal photoreceptor and their released exosomes
	Primary retinal photoreceptor cells isolated from C57BL/6J mice and exposed to high glucose (30 mM) for 96 h, as a model of DR		
Trotta et al., 2020 [40]	In vivo	RvD1 (50 nM) alone or combined with the ALX/FPR2 inhibitor Boc-2 (20 µM) added to primary retinal photoreceptor cells at the time of adding high glucose	Primary retinal photoreceptor cells exhibited short and small mitochondria (fragmentation, aggregation), along with high cytosolic MMP-9, MMP-2 activity. RvD1 (50 nM) restored mitochondrial morphology and function, improved mitochondrial DNA repair and cell survival, by decreasing MMP-9 and MMP-2 activity
	Primary retinal photoreceptor cells isolated from C57BL/6J mice and exposed to high glucose (30 mM) for 96 h, as a model of DR		
Trotta et al., 2021 [38]	In vivo	-	Endogenous retinal levels of RvD1 were reduced in aged mice, with a higher decrement in aged males. RvD1 levels negatively correlated with retinal levels of Iba-1 (microglia activation), GFAP (astrocyte activation), NF-kB and TNF-α (neuroinflammation), caspase 3 (apoptosis), and nitrosative stress.
	Balb-c mice aged 3 months (control group) and 24 months (aged group; approximately 75–85 years for humans), as a model of aged retina		

Abbreviations. O_2_: oxygen; PUFAs: polyunsaturated fatty acids; AA: arachidonic acid; DHA: docosahexaenoic acid; EPA: eicosapentaenoic acid; RvD1: resolvin D1; RvE1: resolvin E1; CNV: choroidal neovascularization; CRECs: choroid-retinal endothelial cells: PMN: polymorphonuclear leukocytes; IL-1β: interleukin 1 beta; h(s): hour(s); VCAM-1: vascular cell adhesion molecule-1; IL-8: interleukin 8; MIP-1β: inflammatory macrophage protein-1 beta; TNF-α: tumor necrosis factor alpha; EIU: endotoxin-induced uveitis; LPS: lipopolysaccharide; ALX/FPR2: lipoxin A4 receptor (ALX)/formyl peptide receptor; SIRT1: sirtuin 1; VEGF: vascular endothelial growth factor; DR: diabetic retinopathy; MMP-9 and MMP2: metalloproteinase matrix metallopeptidase 9 and 2; Iba-1: ionized calcium-binding adapter molecule 1; GFAP: glial fibrillary acidic protein; NF-kB: nuclear factor kappa-light-chain-enhancer of activated B cells.

### 2.3. Protectins

NPD1, also known as 10R,17S-dihydroxy-docosa-4Z,7Z,11E,13E,15E,19Z hexaenoic acid, is a DHA-derived mediator formed by the action of phospholipase A_2_ (PLA_2_)-15-lipoxygenase (15-LOX) [32,47]. At nanomolar concentrations, NPD1 exhibited protective actions from oxidative stress-induced apoptosis in different retinal layers, such as RPE cells, POS cells and retinal ganglion cells (RGCs), by promoting cells’ integrity. 

In this regard, in ARPE-19 cells the activation of NPD1 synthesis was reported as an early response to oxidative stress and was considered a newly recognized function of RPE cells [48,49]. Indeed, by acting on human RPE cells in an autocrine fashion, NPD1 exerts anti-apoptotic effects related to a downregulation of the pro-apoptotic proteins Bax, Bad and caspase 3, along with the reduction of the apoptotic DNA damage and the inhibition of IL-1β stimulated expression of COX-2 [32,50]. This action was evident especially in 15-LOX1-deficient RPE cells, whose survival was selectively rescued by NDP1 [49,51,52]. 

Worthy of note, the binding of NPD1 with RPE cells seems to be stereoselective and highly specific, since neither LXA4 or RvE1 were able to compete for the binding with RPE cells [22]. Interestingly, NPD1 actions can also be exerted in a paracrine mode. NPD1 is able to diffuse through the interphotoreceptor matrix (IPM) and to act on photoreceptor cells and/or Müller cells [50,53]. Specifically, NPD1 is a very important mediator in the POS protection mediated by RPE against oxidative stress [54]. By reducing the POS progressive impairment, NDP1 is able to exert neuroprotective functions during the process of phototransduction [47]. Indeed, NPD1 increased content and bioactivity in POS phagocytosis was considered a distinct signaling able to promote cell integrity and the survival of POS cells [48,53,55]. 

NPD1 was also able to inhibit CNV in a mouse model of CNV induced by laser rupture of Bruch’s membrane: NPD1 protective effects could be due to the inhibition of NF-kB, with the consequent reduction in COX-2 activity and VEGF expression [56]. In an OIR mouse model, NPD1 was able to reduce the vascular loss and pathological regrowth after hypoxic injury [46]. In the same experimental model, the increase of NPD1 levels by the oral administration of the neutraceutical arginine-glutamine (Arg-Glu) resulted in a restoration of the vascular density of the retina [57]. 

Overall, NPD1 synthesis and bioactivity seem to promote retinal cell survival in oxidative stress conditions, potentially reducing the visual system impairment in retinal degenerative diseases (Table 3). Therefore, a deeper knowledge of NPD1 signaling and actions may lead to the development of new therapeutic strategies in retinopathy management. 

### 2.4. Maresins

MaRs are SPMs synthetized by macrophages from DHA through 12-LOX and enzymatic hydrolysis, with marked anti-inflammatory and pro-resolving effects in LPS-acute lung injury and experimental colitis mouse models, mechanic allodynia and chemotherapy-induced neuropathic pain in mice, human endothelial and vascular smooth cells, diabetic nephropathy, and tissue rigeneration in planaria after *Escherichia* coli infection [20]. By focusing on ocular pathologies, MaR1 has recently been shown to maintain optimal tear film mucin levels in cultured rat conjunctival goblet cells, by stimulating mucin secretion [59]. To our knowledge, no studies investigating the involvement of MaRs in retinopathies have been performed until now, although MaRs are considered, promising SPMs for the management of all inflammatory ocular pathologies [1].

### 2.5. Annexins

**Figure 2:**
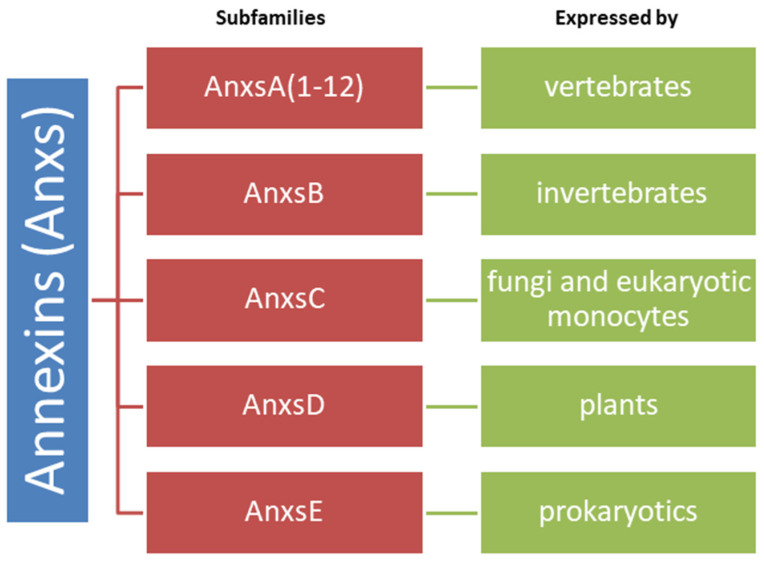
Annexins categories and their expression in different phyla.

The annexins (Anxs) constitute a family of more than 60 highly conserved proteins, characterized by a conserved structural element, the so-called Anx repetition, and are able to bind negatively charged phospholipid in a calcium-dependent manner [60]. They are divided into five categories, with a differential cellular expression: AnxsA (12 subtypes), AnxsB, AnxsC, AnxsD and AnxsE [61] (Figure 2).

AnxA1 is another among the PMs, but overall, all Anxs are able to modulate apoptosis and neoangiogenesis [25].

AnxA1 and its receptor ALX/FPR2 are constitutively expressed in both healthy mouse and human retina, with a specific localization of AnxA1 in RGCs and RPE cells, and a FPR2 positive staining in ganglion cell layer (GCL), ONL, and POS [62]. Both AnxA1 and ALX/FPR2 receptor resulted increased in ARPE-19 cells exposed to hypoxic conditions [63]. It is worth noting that, in different in vitro and in vivo models of CNV, hypoxic ARPE-19 cells were able to secrete higher levels of AnxA1, which had protective effects on human choroidal endothelial cells’ (HCECs) functions, by promoting their proliferation, tube formation and migration. Interestingly, NLR Family Pyrin Domain Containing 3 (NLRP3)-inflammasome activation and, consequently, NLRP3-inflammosome mediated pyrocitosis, were markedly reduced by AnxA1 in HCECs, resulting in an overall reduction of CNV volume [63]. Therefore, AnxA1 could be considered a new target for CNV prevention and management.

Regarding AnxA2, there are controversial data describing its role in the genesis of retinal neovessels [64]. For example, in an OIR mouse model, which mimics many aspects of human PDR, the normal neovascular response of endothelial cells was associated with high levels of AnxA2. Indeed, in wild type mice, endothelial cells overexpressing AnxA2 were found able to react to oxygen stimulus, by migrating from the retina to the inner limiting membrane (ILM), and then by penetrating ILM. On the contrary, the absence of AnxA2 showed a severely impaired angiogenic response to oxygen stimulation (−50%), with endothelial cells failing to migrate to the ILM in AnxA2-null mice [65]. However, in the same mouse model of oxygen-induced retinal neovascularization, the overexpression of AnxA2 was reported by Wang et al. as a promoter of pathological retinal neoangiogenesis, whose inhibition could be a novel strategy to counteract retinal neovascularization [66]. This evidence was also supported by the results obtained in a mouse model of ischemia-induced retinal neovascularization, in RPE cells silenced for AnxA2 and exposed to photocoagulation and in a rat model of argon laser coagulation-induced CNV, in which AnxA2 emerged as an inducer of the VEGF-VEGF receptor 2 (VEGFR2) pathway [67,68].

Some authors have also examined the expression of the anti-inflammatory protein AnxA1 in mice and the human retina during uveitis. They also investigated whether local administration of recombinant human AnxA1 (hrAnxA1) can suppress uveitis in mice. The authors observed that retinal expression of AnxA1 increased during uveitis compared with healthy controls. AnxA1 predominantly colocalizes with cells expressing CD45, microglia positive to glial fibrillary acidic protein (GFAP) and, to a lesser extent, Ionized Calcium-binding Adapter Molecule 1 (Iba-1) positive myeloid cells. Finally, local treatment with hrAnxA1 attenuated the severity of uveitis in mice by reducing the local expression of pro-inflammatory cytokines and chemokines. These data provide proof of concept for a novel therapeutic approach for the treatment of patients with non-infectious uveitis [62].

Therefore, due to their properties in the modulation of neoangiogenesis and inflammation (Table 4), Anxs are intriguing molecules in the field of ophthalmology, and particularly for DR and uveitis progression.

## 3. Perspectives on New Non-Lipidic Mediators in Retinopathies and Specific Tools

PMs, mainly formed by 12-LOX and 15-LOX, can be generated in the retina and supported in their action by non-lipidic mediators that would have been present in the retina, for example, galectins. They could be released following stimulation of inflammatory cells by galectin-1 (Gal-1) [70], although a clear role of galectins in retinopathies warrants further deepening. Indeed, the pathogenesis of common retinal disorders has been recently associated with the modulation of galectins [71]. These are β-galactoside-binding proteins involved in the regulation of vascular permeability and angiogenesis, neuroinflammation and oxidative stress, all contributing to the progression of different retinopathies [71]. Particularly, higher Gal-1 plasmatic levels were found in diabetic patients [72], while retinal Gal-1 over-production was found in diabetic rodent models [72,73] and has a key role in the progression of retinal fibrosis [74]. Similarly, galectin 3 (Gal-3) plasma levels were elevated in patients with type 2 diabetes [75], whereas the Gal-3(−/−) -induced diabetes mice model showed significantly less retinal pathology compared to controls [76]. Thus, Gal-1 and Gal-3 are intimately involved in regulating diabetic-associated processes, including retinal dysfunction, and attention should be focused on finding molecular targets that block the production of these proteins.

Several antagonists for Gal-1 and Gal-3 have recently been developed, some of which are in clinical trials that may be successful from the perspective of inflammatory retinal disorders. For example, TD139 was found to suppress Gal-3 expression on bronchoalveolar lavage (BAL) macrophages and decrease the plasma biomarkers associated with idiopathic pulmonary fibrosis progression [77] or hyperinflammation in Coronavirus Disease 2019 (COVID-19) [78]. Recently, OTX008, a calixarene derivative, has recently been shown to inhibit Gal-1 overproduction in hyperglycemic conditions. It was found that transcription factor adaptor protein complex 4 (AP-4) induced overproduction of Gal-1 under hyperglycemia; OTX008 blocks the activation of the Akt serine threonine kinase pathway and prevents the accumulation of Gal-1 [79], suggesting a novel role as a Gal-1 inhibitor and a possible therapeutic target to treat fibrosis in diabetes complications.

## 4. Conclusions

Inflammation is a key element in the pathological progression of the most common retinal diseases. Its resolution by well-known PMs will undoubtedly contribute to improving the prevention and treatment of AMD, uveitis and DR. Nevertheless, to the author’s knowledge, a better understanding of the molecular pathways to be deactivated in the retina, such as the Gal-1 pathway, is an important goal for clinical management by means of novel compounds.

## Figures and Tables

**Table 1:** Lipoxins and retinal damage.

Author, Year	Model	Treatment	Main Results
Medeiros et al., 2008 [31]	In vivo	Topical administration (20 µL eye drops) of LXA4 1, 5, or 10 ng/eye to both eyes, 1 h before LPS and 6–12–18 h after LPS	Topical LXA4 (1–10 ng/eye) pre-treatment and 10 ng/eye post-treatment reduced inflammatory cell number and the protein leakage into the aqueous humor. IL-1, PGE2, VEGF, COX-2 and TNF-α in LXA4 treated eyes were reduced
	EIU in male Wistar rats, induced with 200 µg of LPS into one rat hind paw		
Kaviarasan et al., 2015 [28]	Case control study	-	LXA4 levels were significantly decreased in NPDR and PDR patients
	27 healthy controls, 27 diabetic patients without retinopathy, 30 NPDR and 30 PDR patients		
	In vitro	LXA4 (10, 25, 50 nmol/L) treatment 30 min before LPS	LXA4 reduced IL-6 at lower concentrations
	ARPE-19 cells exposed to LPS (500 ng/mL) for 48 h		
Lu et al., 2019 [29]	In vivo	Intravitreal injection of LXA4 (100 ng/μL) at 6, 9 and 12 postnatal days	LXA4 intravitreal injections delay the loss of visual function, by reducing POS apoptosis and inhibiting microglial activation
	RD1 mice as a model of rapid retinal degeneration		
Xie et al., 2021 [30]	In vivo	Mice were orally administered with LXA4 once a day for 3 days before blue-light exposure	LXA4 treatment reduces ONL degeneration and improved the number of cells positive to zonula occludens-1 staining
	Male Balb-c mice exposed to blue-light (10,000 lux, 430 nm) for 1 h/day for 14 days, as a model of retinal degeneration		
	In vitro	ARPE-19 cells were treated with LXA4 (50, 100 nM) 30 min before blue-light exposure	LXA4 treatment reduced cell death and ROS content in RPE cells by activating NRF2/HO1 pathway
	ARPE-19 cells exposed to blue-light illumination (1000 lux, 430 nm) for 15 h		

Abbreviations. LXA4: lipoxin A4; EIU: endotoxin-induced uveitis; LPS: lipopolysaccharide; h(s): hour(s); IL-1: interleukin 1; PGE2: prostaglandin E2; VEGF: vascular endothelial growth factor; COX-2: cyclooxygenase 2; TNF-α: tumor necrosis factor alpha; NPDR: non-proliferative diabetic retinopathy; PDR: proliferative diabetic retinopathy; ARPE-19: adult retinal pigment epithelial cell line-19; IL-6: interleukin 6; POS: photoreceptor outer segment; ONL: outer nuclear layer; ROS: reactive oxygen species; RPE: retinal pigment epithelium; NRF2/HO1: nuclear factor erythroid 2–related factor 2/Heme Oxygenase 1.

**Table 3:** NPD1 and retinal damage.

Author, Year	Model	Treatment	Main Results
Mukherjee et al., 2004 [32]	In vitro	NPD1 (50 nM) added to ARPE-19 cells at the time of adding TNF-α/H_2_O	NPD1 inhibited ARPE-19 apoptosis induced by oxidative stress
	RPE-19 cells serum- starved and exposed to oxidative stress for 14 h (400–800 µM H_2_O_2_ and 10 ng/mL TNF-α)		
	ARPE-19 cells transfected with COX-2 promoter	NPD1 (0.05–0.5–5–50 100 nM) added to ARPE-19 cells	NPD1 reduces IL-1β content induced by COX2
Connor et al., 2007 [46]	In vivo	C57BL/6J mice fed with diet containing 2% ω-6-PUFAs (AA) and no ω-3-PUFAs (DHA and EPA), or 2% ω-3-PUFAs and no ω-6-PUFAs in 10% (*w/w*) safflower oil	Among the bioactive ω-3-PUFA-derived mediators, NPD1 potently protected against neovascularization
	C57BL/6J mice exposed to 75% O_2_ from postnatal day 7 to postnatal day 12, then returned to room air for 5 days as a model of hypoxia-induced ischemic retinopathy		
Mukherjee et al., 2007 [58]	In vitro		
	ARPE-19 cells serum-starved and exposed to: - A2E, a lipofuscin component (20 μM) in the presence of 430 nM light and O_2_ for 15 min, followed by 60 min of incubation in the dark, as a model of oxidative stress; - 600 µM H_2_O_2_ and 10 ng/mL TNF-α) for 15 h, as a second model of oxidative stress	NPD1 (50 nM) added 5 min before oxidative stress and at different time points after oxidative stress	NPD1 decreased ARPE-19 damage in both models of oxidative stress
		DHA (30 nM) and PEDF (20 ng/mL) added to ARPE-19 cells at the time of adding TNF-α/H_2_O_2_	PEDF increased NPD1 synthesis and release through the apical surface of ARPE-19 cells. PEDF-induced NPD1 synthesis and release is selectively potentiated by DHA
Mukherjee et al., 2007 [53]	In vitro	ARPE-19 cells incubated with bovine POS (10 million per well)	NPD1 increased in POS-mediated ARPE-19 protection against oxidative stress
	ARPE-19 cells serum-starved and exposed to oxidative stress (H_2_O_2_ and 10 ng/mL TNF-α) for 16 h		
Calandria et al., 2009 [49]	In vitro		
	ARPE-19 cells silenced for 15-LOX, then serum-starved and exposed to oxidative stress (30% H_2_O_2_ and 10 ng/mL TNF-α) at different time points	ARPE-19 cells exposed to 50 nM NPD1 after oxidative stress	NPD1 not detectable in 15-LOX silenced-ARPE-19 cells
			NPD1 promoted cell survival in 15-LOX silenced cells, by reducing oxidative stress
Marcheselli et al., 2010 [22]	In vitro	NPD1 50 nM added to ARPE-19 cells at the time of adding TNF-α/H_2_O_2_	NPD1 showed a specific and stereoselective binding capacity to ARPE-19 cells and inhibited cell apoptosis induced by stress induction
	ARPE-19 cells serum- starved and exposed to oxidative stress (600 μM H_2_O_2_ and 10 ng/mL TNF-α) for 15 h		
Sheets et al., 2010 [56]	In vivo	Intraperitoneal injections of NPD1 (1 mg/mL) 1 h before laser treatment and 1, 3, 5 and 7 days after laser treatment	NPD1 reduces CNV lesions and cell proliferations at lesion sites
	Male C57BL/6J mice receiving laser rupture of Bruch’s membrane as a model of CNV		
Shaw et al., 2018 [57]	In vivo	Arg-Gln dipeptide (1, 2.5 or 5 g/kg body weight per day on postnatal day 12 and 17	Arg-Gln dipeptide restored retinal DHA and NPD1 levels in OIR model, by reducing preretinal neovascularization and vaso-obliteration
	7-day-old C57BL/6J mice exposed to 5% oxygen atmosphere for 5 days as a model of OIR		

Abbreviations. RPE: retinal pigment epithelium; H_2_O_2_: hydrogen peroxide; TNF-α: tumor necrosis factor alpha; ARPE-19: adult retinal pigment epithelial cell line-19; COX-2: cyclooxygenase 2; NPD1: neuroprotectin 1; IL-1β: interleukin 1 beta; O_2_: oxygen; PUFAs: polyunsaturated fatty acids; AA: arachidonic acid; DHA: docosahexaenoic acid; PEDF: pigment epithelium-derived factor; EPA: eicosapentaenoic acid; h(s): hour(s); POS: photoreceptor outer segment; LOX: lipoxygenase; CNV: choroidal neovascularization; OIR: oxygen-induced retinopathy.

**Table 4:** Annexins and retinal damage.

Author, Year	Model	Treatment	Main Results
Ling et al., 2004 [65]	In vivo	-	AnxA2-null mice showed an impaired angiogenic response to O_2_ stimulation (-50%), with endothelial cells failing to react to O_2_ by not migrating to the ILM and penetrating in the same retinal layer
	AnxA2-null C57BL/6 mice, exposed at postnatal day 7 to continuous-flow 75% O_2_/25% N_2_ for 5 days, then returned to room air		
Zhao et al., 2009 [67]	In vivo	Intravitreous injection of AnxA2 (1 μg) or AnxA2 sirna (1 μg) at postnatal day 7 and 12 in mice returned to room air.	AnxA2 increased in endothelial cells of ischemic retina. More neovascularization in eyes injected with AnxA2 than in eyes injected with AnxA2 sirna.
	C57BL/6J mice exposed to 75% O_2_ from postnatal day 7 to postnatal day 12, then returned to room air for 5 days as a model of hypoxia-induced ischemic retinopathy		
	In vitro	25 ng/mL VEGF for 2 h	AnxA2 upregulated by VEGF; high expression of VEGFR2 consequent to AnxA1 increase
	CRECs exposed to hypoxic conditions (1% O_2_, 94% N_2_, 5% CO_2_) for 2 and 4 h		
Zhao et al., 2020 [68]	In vivo	-	AnxA2 and VEGF showed the maximum expression after 14 days from CNV in vascular endothelial cells, RGCs, INL and RPE cells
	Norvegian rats exposed to argon laser coagulation-induced CNV		
	In vitro		VEGF expression was reduced by the silenced expression of AnxA2
	RPE-J cells silenced for AnxA2 and exposed to photocoagulation by laser ablation		
Wang et al., 2013 [66]	In vivo	AnxA2 siRNA	AnxA2 overexpressed in OIR mice, paralleled by high neovessel growth. VEGF, metalloproteinases 2 and 9 (MMP-2 and MMP-9) reduced by AnxA2 siRNA
	C57BL/6 mice, exposed to OIR		
Davis et al., 2017 [69]	In vivo	-	RGCs showing apoptosis in OHT animals were positive to Anx5 staining
	Adult male Dark Agouti rats injected with hypertonic saline solution (1.80 M) into two episcleral veins as a model of OHT for 21 days		
Gardner et al., 2017 [62]	In vivo	Intravitreal injections (2 µL) of hrAnxA1 (50 and 500 ng) in EIU mice	AnxA1 and ALX/FPR2 receptor are expressed in healthy mice and human retina (AnxA1 in RGCs and RPEs cells; ALX/FPR2 in GLC, ONL and POS) AnxA1 predominantly colocalizes with cells positive to CD45, GFAP and Iba-1
	EIU in C57BL/6 mice, induced with 1 ng LPS intreavitreally injected (2 µL)		
	Post-mortem human retinae from healthy patients or patients with uveitis	-	hrAnxA1 (500 ng) attenuated the EIU severity in mice by reducing the expression of pro-inflammatory cytokines and chemokines
Zhu et al., 2022 [63]	In vitro	HCECs exposed to AnxA1 recombinant protein	ALX/FPR2 expression, along with AnxA1 expression and secretion are upregulated by hypoxia in ARPE-19 cells. AnxA1 promoted HCECs proliferation, migration and tube formation. Moreover, AnxA1 inhibited NLRP3-inflammosome activation and NLRP3-inflammasome mediated pyrocitosis in HCECs
	ARPE-19 cells and HCECs exposed to hypoxic conditions (1% O_2_, 5% CO_2_ and 94% N_2_) for 24 h as a model of CNV		
	In vivo		AnxA1 secreted from RPE cells resulted in an overall reduction of CNV volume
	male C57BL/6J mice were subjected to laser photocoagulation (rupture of Bruch’s membrane) as a model of CNV		

Abbreviations. AnxA2: annexin A2; O_2_: oxygen; N_2_: nitrogen; ILM: inner limiting membrane; CRECs: choroid-retinal endothelial cells; CO_2_: carbon dioxide; h(s): hour(s); VEGF: vascular endothelial growth factor; VEGFR2: vascular endothelial growth factor receptor 2; AnxA1: annexin A1; CNV: choroidal neovascularization; RGCs: retinal ganglion cells; INL: inner nuclear layer; RPE: retinal pigment epithelium; OIR: oxygen-induced retinopathy; MMP-9 and MMP2: metalloproteinase matrix metallopeptidase 9 and 2; OHT: ocular hypertension; Anx5: annexin 5; EIU: endotoxin-induced uveitis; LPS: lipopolysaccharide; ALX/FPR2: lipoxin A4 receptor (ALX)/formyl peptide receptor; GLC: ganglion cells; ONL: outer nuclear layer; POS: photoreceptor outer segment; GFAP: glial fibrillary acidic protein; Iba-1: ionized calcium-binding adapter molecule 1; ARPE-19: adult retinal pigment epithelial cell line-19; HCECs: human choroidal endothelial cells; NLRP3: NLR Family Pyrin Domain Containing 3.

## Data Availability

Not applicable.
